# Multidrug-Resistant ESBL/AmpC-Producing *Klebsiella pneumoniae* Isolated from Healthy Thoroughbred Racehorses in Japan

**DOI:** 10.3390/ani10030369

**Published:** 2020-02-25

**Authors:** Eddy Sukmawinata, Ryoko Uemura, Wataru Sato, Myo Thu Htun, Masuo Sueyoshi

**Affiliations:** 1 Graduate School of Medicine and Veterinary Medicine, University of Miyazaki, Miyazaki 889-1692, Japan; eddyswinata@gmail.com (E.S.); a0d802u@cc.miyazaki-u.ac.jp (M.S.); 2 Department of Veterinary Sciences, Faculty of Agriculture, University of Miyazaki, Miyazaki 889-2192, Japan; wataru9356@gmail.com (W.S.); drmyothu.htun@gmail.com (M.T.H.); 3 Center for Animal Diseases Control, University of Miyazaki, Miyazaki 889-2192, Japan

**Keywords:** extended-spectrum β-lactamase, AmpC β-lactamase, *Klebsiella pneumoniae*, horse, multidrug resistance

## Abstract

**Simple Summary:**

Extended-spectrum β-lactamases (ESBLs) and AmpC β-lactamases (AmpCs) have been recognized as an emerging global problem in humans and animals. These enzymes provide a mechanism of resistance by inactivating β-lactam antibiotics and are mostly encoded on plasmids, which can be easily transmitted to other bacteria in humans, animals, and the environment. Several clinical diseases caused by *Klebsiella* spp. infection have been confirmed in the horse community. The emergence of antimicrobial resistance in *Klebsiella* spp. increases the risk of treatment failure in infected horses. In this study, we investigated the presence of ESBL/AmpC-producing *Klebsiella* spp. isolated from healthy Thoroughbred racehorses in Japan. The results showed that ESBL/AmpC-producing *Klebsiella pneumoniae* (ESBL/AmpC-KP) isolated from horses have co-resistance to other β-lactam antibiotics as multidrug-resistant (MDR) bacteria. Genetic relatedness analysis suggested that plasmid-mediated AmpC-KP clones may spread between horses. This is the first study to show *K. pneumoniae* carrying MDR plasmid-mediated AmpC isolated from racehorses. Continuous monitoring antimicrobial resistance to this species is required in order to control the spread of MDR ESBL/AmpC-KP in the racehorse community.

**Abstract:**

Extended-spectrum β-lactamase (ESBL)- and AmpC β-lactamase (AmpC)-producing *Klebsiella* spp. have become a major health problem, leading to treatment failure in humans and animals. This study aimed to evaluate the presence of ESBL/AmpC-producing *Klebsiella* spp. isolated from racehorses in Japan. Feces samples from 212 healthy Thoroughbred racehorses were collected from the Japan Racing Association Training Centers between March 2017 and August 2018. ESBL/AmpC-producing *Klebsiella* spp. were isolated using selective medium containing 1 µg/mL cefotaxime. All isolates were subjected to bacterial species identification (MALDI-TOF MS), antimicrobial susceptibility test (disk diffusion test), characterization of resistance genes (PCR), conjugation assay, and genetic relatedness (multilocus sequence typing/MLST). Twelve ESBL/AmpC-producing *Klebsiella pneumoniae* (ESBL/AmpC-KP) were isolated from 3.3% of horse samples. Antimicrobial resistance profiling for 17 antimicrobials showed all ESBL/AmpC-KP were multidrug-resistant (MDR). Only 1 isolate was confirmed as an ESBL producer (*bla*_CTX-M-2-_positive), whereas the other 11 isolates were plasmid-mediated AmpC (pAmpC) producers (*bla*_CMY_ positive). On the basis of MLST analysis, the ESBL-KP isolate was identified as sequence type (ST)-133 and four different STs among AmpC-KP isolates, ST-145, ST-4830, ST-4831, and ST-4832, were found to share six of the seven loci constituting a single-locus variant. This is the first study to show *K. pneumoniae* carrying MDR pAmpC isolated from a racehorse.

## 1. Introduction

*Klebsiella* spp. is a normal intestinal bacteria in horses [1] and is ubiquitous in the environment [2]. However, some studies have reported *Klebsiella* spp. as a causal agent for infections in horses, such as mares with metritis and cervicitis, foals with septicemia and pneumonia [3], and pneumonia in adult horses [2], and disease severity depends on the pathogenicity of the strains [1]. In the horse industry, about 25%–60% of economic losses are caused by endometritis, and *Klebsiella pneumoniae* was reported as one of the causal infections that can be transmitted through the venereal route [4,5]. First-, second-, and third-generation cephalosporin has been used for treatment of bacterial infection in equine medicine for several years. Ceftiofur, which belongs to third-generation cephalosporin, is approved for used in horses and effective in treatment of *Klebsiella* infection. In special cases, such as septicemia in foals and respiratory tract disease in horses, cefquinome, which is a fourth-generation cephalosporin, is accepted for use in the United Kingdom [6]. However, the occurrence of antimicrobial resistance (AMR) in *Klebsiella* spp. has increased the risk of treatment failure [7].

Extended-spectrum β-lactamases (ESBLs) and AmpC β-lactamases (AmpCs) have emerged globally in humans and animals [8]. These enzymes can hydrolyze extended-spectrum cephalosporin [8], whereas AmpCs have a broader resistance spectrum to cephalosporins, including cephamycins (cefoxitin and cefotetan) [9]. ESBL and AmpC genes are mainly located on mobile genetic elements such as plasmids, which can be transferred to other bacteria in humans, animals, or the environment [10]. Nonetheless, AmpC is less frequently reported than ESBL [11,12]. β-Lactamase inhibitors such as clavulanic acid, sulbactam, and tazobactam have the effect of inhibiting the production of ESBL [9,13], but these have much less effect on AmpC β-lactamase [12]. 

Extended-spectrum β-lactamase-producing *Enterobacteriaceae* have gained special attention on AMR in horses due to their presence as a potentially zoonotic bacteria [14]. The CTX-M family of ESBL have been reported as the predominant type of ESBL after the TEM and SHV types [15], and more than 200 CTX-M variants have been identified worldwide [16]. On the other hand, some species of *Enterobacteriaceae* (such as *Enterobacter cloacae*, *Enterobacter aerogenes*, *Aeromonas* sp., *Citrobacter freundii*, *Providencia* sp., *Serratia marcescens*, *Hafnia alvei*, *Morganella morganii*, and *Pseudomonas aeruginosa*) have resistance to extended-spectrum cephalosporin, which may be caused by inducible chromosomal AmpC. Furthermore, plasmid-mediated AmpC (pAmpC) were identified from *Enterobacteriaceae* such as, *Klebsiella* spp., *Escherichia coli*, *Salmonella* spp., and *Proteus mirabilis* [17]. The distribution of pAmpC seems to be more frequent in animals than in humans [16]. Although ESBL/AmpC-producing *Klebsiella* spp. (ESBL/AmpC-K) are considered a major global concern, information is still lacking for AMR in horses [1]. Moreover, information on ESBL/AmpC-K in horses is unavailable in Japan. This study aimed to evaluate the presence of ESBL/AmpC-K isolated from healthy Thoroughbred racehorses in Japan. In addition, although carbapenems are rarely used in pet animals, these antimicrobials are frequently considered as the last option of treatment for ESBL/AmpC-producing bacteria infection [18,19]. In this work, all ESBL/AmpC-positive isolates were also tested for carbapenemase production. 

## 2. Materials and Methods

### 2.1. Isolation of ESBL/AmpC-K

Feces samples from 212 healthy Thoroughbred racehorses were collected by veterinarians at the Japan Racing Association (JRA) between March 2017 and August 2018. Sampling locations were the Miho Training Center (103 samples) and Ritto Training Center (109 samples). No samples were from horses under treatment with antibiotics. Fresh feces samples from each individual horse were collected and stored in sterile plastic bags. Samples were sent immediately to our laboratory in a cooling box. ESBL/AmpC-K was screened on the basis of the European Committee on Antimicrobial Susceptibility Testing (EUCAST) guideline by using MacConkey agar (Nissui Pharmaceutical Co., Ltd., Tokyo, Japan) supplemented with 1 µg/mL cefotaxime (CTX; Duchefa Biochemie B.V. Haarlem, North Holland, the Netherlands) [20]. One to three colonies with pink, mucoid, and lactose fermented appearance were selected for species identification by using MALDI-TOF MS (Bruker, Billerica, MA, USA). All presumptive ESBL/AmpC-K isolates were stored frozen in trypticase soy broth (Nissui Pharmaceutical Co., Ltd., Tokyo, Japan) with 20% glycerol at −80 °C for further analysis. *Klebsiella pneumoniae* ATCC 700603 and *E. coli* ATCC 25922 were used as positive and negative control type strains, respectively.

All presumptive isolates were confirmed for ESBL and AmpC production by using the AmpC and ESβL Detection Set (D68C). All ESBL/AmpC positive isolates were further tested for carbapenemase production by Mastdiscs Combi Carba Plus (D73C), and the results were interpreted based on manufacturer guidelines (Mast Diagnostics, Merseyside, United Kingdom).

### 2.2. Antimicrobial Susceptibility Test

The antimicrobial susceptibility testing of all isolates were performed by disk diffusion assay to 17 antimicrobial agents belonging to 8 classes of antimicrobial, β-lactam (ampicillin 10 μg (ABPC), cefuroxime 30 μg (CXM), cefotaxime 30 μg (CTX), ceftazidime 30 μg (CAZ)), aminoglycoside (gentamicin 10 μg (GM), kanamycin 30 μg (KM), streptomycin 10 μg (SM), tetracycline (tetracycline 30 μg (TC), oxytetracycline 30 μg (OTC), doxycycline 30 μg (DOXY)), amphenicol (chloramphenicol 30 μg (CP)), polypeptide (colistin 10 μg (CL)), quinolone (nalidixic acid 30 μg (NA), norfloxacin 10 μg (NFLX), marbofloxacin 5 μg (MAR)), fosfomycin 200 μg (FOM), and folate antagonist-sulfonamide (trimethoprim/sulfamethoxazole 1.25/23.75 μg (STX)). Minimum inhibition zones were interpreted using the Clinical Laboratory Standard Institute (CLSI) criteria [21]. Multidrug-resistant (MDR) bacteria were termed to isolates that had resistance to at least three or more classes of antimicrobials [22]. *E. coli* ATCC 25922 strain was used for quality control.

### 2.3. Molecular Characterization of ESBL/AmpC-K

DNA from ESBL/AmpC-K isolates was extracted on the basis of the previously described method [23]. All ESBL/AmpC-positive isolates, the CTX-M-type β-lactamase and pAmpC genes were detected by multiplex PCR [24,25]. The *bla*_TEM_ and *bla*_SHV_ genes were identified by PCR and directly sequenced to confirm the type of β-lactamase [24]. Chromosomal AmpC, *bla*_CMY_, *strA*, *strB*, *aphA1*, *tetA*, *tetB*, *cat*, and *floR* genes were identified by PCR [26,27,28], then one positive sample for each gene was selected for DNA sequencing to confirm the expected size, which was used as a positive control for other samples [24]. The results were analyzed with MEGA 7.0 (https://www.megasoftware.net/) and were examined with the National Center for Biotechnology Information, Basic Local Alignment Search Tool (NCBI BLAST) program (http://www.ncbi.nlm.nih.gov/blast/). The sequence types (STs) of *K. pneumoniae* were identified by multilocus sequence typing (MLST) on the basis of a previous report [29]. Novel STs were submitted to *Klebsiella pneumoniae* PubMLST and were termed as new STs (https://bigsdb.pasteur.fr/klebsiella/klebsiella.html).

### 2.4. Conjugation Assay

Transfer of antibiotic resistance was studied using conjugation for all ESBL/AmpC-K isolates. A plasmid-free and nalidixic acid-resistant (F^−^, Na^r^) of *E. coli* DH5α (Takara Bio Inc., Shiga, Japan) was used as a recipient strain, whereas all ESBL/AmpC-K resistant to NA served as donors. Conjugation was performed on the basis of our previous study [24].

### 2.5. Statistical Analysis

The antimicrobial susceptibility profile and the efficiency of conjugation were analyzed by descriptive statistics using Excel 2017 (version 15.40; Microsoft, Redmond, WA, USA).

## 3. Results

### 3.1. Resistance Phenotype

**Figure 1:**
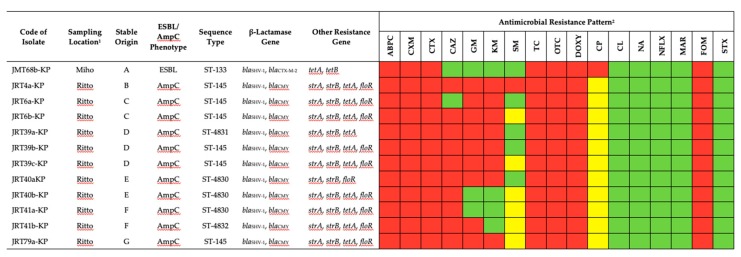
Characteristics of extended-spectrum β-lactamase/AmpC β-lactamase-producing *Klebsiella pneumoniae* (ESBL/AmpC-KP) isolated from Thoroughbred racehorses in Japan. ^1^ Miho, Miho Training Center, Japan Racing Association, Ibaraki; Ritto, Ritto Training Center, Japan Racing Association, Shiga. ^2^ ABPC, ampicillin; CXM, cefuroxime; CTX, cefotaxime; CAZ, ceftazidime; GM, gentamicin; KM, kanamycin; SM, streptomycin; TC, tetracycline; OTC, oxytetracycline; DOXY, doxycycline; CP, chloramphenicol; CL, colistin; NA, nalidixic acid; NFLX, norfloxacin; MAR, marbofloxacin; FOM, fosfomycin; STX, trimethoprim/sulfamethoxazole; red, resistance; yellow, intermediate; green, susceptible.

In this study, 12 ESBL/AmpC-producing *K. pneumoniae* (ESBL/AmpC-KP) were isolated from 7 (3.3%; 7/212) healthy Thoroughbred racehorse feces samples. Phenotypically, 11 isolates were confirmed as AmpC producers from 6 samples (2.8%; 6/212) that came from the Ritto Training Center, and only 1 sample from the Miho Training Center was confirmed as an ESBL producer. All ESBL/AmpC isolates were not identified as carbapenemase producers. All samples were resistant to ABPC, CXM, CTX, TC, OTC, DOXY, and FOM, followed by CAZ (83.3%; 10/12), GM (75.0%; 9/12), KM (66.7%; 8/12), SM (8.3%; 1/12), and CP (8.3%; 1/12). All isolates (100%; 12/12) were defined as MDR, meaning that they were resistant to at least three classes of antimicrobial. ESBL, AmpC, and resistance phenotype patterns are shown in Figure 1.

### 3.2. Molecular Characteristic of ESBL/AmpC-KP

The presence of SHV-1 β-lactamase genes (non-ESBL) were detected in all (100%; 12/12) ESBL/AmpC-KP isolates, and CTX-M-2 was only detected from one isolate that showed an ESBL phenotype. None of the isolates were positive for chromosomal AmpC genes, whereas CMY, which belonged to the CIT family of pAmpC, was detected in all AmpC phenotype isolates. The *strA*, *strB*, and *tetA*, genes were detected in nearly all (91.7%; 11/12) ESBL/AmpC-KP isolates, followed by *floR* (75.0%; 9/12) and *tetB*, which was only detected in one isolate (8.3%; 1/12). All isolates were subjected to MLST analysis. As a result, ESBL-KP isolate was identified as ST-133, and four different STs among AmpC-KP isolates, ST-145 (54.5%; 6/11), ST-4830 (27.3% 3/11), ST-4831 (9.1%; 1/11), and ST-4832 (9.1%; 1/11), shared six of the seven loci constituting a single-locus variant (SLV). In these results, AmpC-KP ST-4830, ST-4831, and ST-4832 were termed as new STs. Characteristic ESBL/AmpC-KP and other resistance genes are summarized in Figure 1.

### 3.3. Conjugation Assay

Conjugation assay was only successful in ESBL-KP ST-133. Horizontal transmission was confirmed by detection of *bla*_CTX-M-2_ in the transconjugant strain with the frequency of transfer 2 × 10^−4^ per donor cell.

## 4. Discussion

In this study, 3.3% of samples from racehorse feces were confirmed as having ESBL/AmpC-KP. Interestingly, 91.7% of total isolates were AmpC producers, which were only isolated from the Ritto Training Center. One isolate (8.3%) was identified as ESBL-KP, derived from the Miho Training Center. ESBL-KP isolated from horses was reported at 0.2% (3/1347) in the Netherlands [13]. In Germany and other European countries, 3.1% (5/160) of ESBL-KP was reported among clinical horse samples [30]. Another study showed that ESBL-KP was isolated from 1.8% (1/55) of foals on admission to hospital, and the shedding rate increased during hospitalization in Israel [31]. The selection of ESBL producers among *Enterobacteriaceae* is expected as the impact of cephalosporin antibiotics used for medical treatment in horses [24].

In our results, all ESBL/AmpC-KP isolates were detected as carrying *bla*_SHV-1_, which is resistant to penicillin and early generation cephalosporin but not resistant to third-generation cephalosporin. SHV-1 is mainly reported in *K. pneumoniae* and may be due to the gene encoded SHV-1, which was located on the chromosome of this species. SHV-1 β-lactamase has also been reported for up to 20% of plasmid-mediated ampicillin *K. pneumoniae* [32]. Our study also confirmed that ESBL-KP isolate was carried the *bla*_CTX-M-2_ gene. CTX-M-2-producing *E. coli* were also detected from the same horse feces sample (data not shown), as reported in our previous study [33]. Conjugation assay showed that *bla*_CTX-M-2_ was transferred with the frequency of transfer 2 × 10^−4^ per donor cell. This finding suggests that horizontal transmission among bacterial species in horse intestine occurred. In Japan, CTX-M-2-producing *K. pneumoniae* have been confirmed in dogs [7], humans [34,35], and broiler chickens [36]. In addition, conjugative plasmids carrying *bla*_CTX-M-2_ have been reported in *K. pneumoniae* isolated from dairy cows with clinical mastitis [37]. In contrast to ESBL-KP, the presence of AmpC-KP in horses is less well documented, but our study identified them as a dominant β-lactamase producer. 

The screening test for detection of AmpC-producing bacteria can be performed by the same protocol for ESBL screening test, and multiplex PCR has been developed to identify pAmpC [17]. All AmpC phenotype isolates in our study contained *bla*_CMY_ belonging to the *bla*_CIT_ type of the pAmpC gene. CMY-2 is prevalent among AmpC enzymes in the animal sector [38]. None of the pAmpC-KP isolates were conjugative under our experimental conditions. To our knowledge, no previous studies have been published describing the rate of *K. pneumoniae* carrying pAmpC isolated from horses. Plasmid-mediated AmpC has been reported worldwide from enterobacteria not predicted to produce AmpC β-lactamases [12]. In equine medicine, previous studies have shown that pAmpC genes belonging to *bla*_CMY-2_ were detected from extended-spectrum cephalosporin-resistant (ESCR) *E. coli* isolated from diseased horses in the Netherlands (0.1%; 1/1347) and the United Kingdom (3.8%; 2/52) [13,39]. The *bla*_CMY_ was also identified from *Salmonella* spp. isolated from horses in the United States and Ireland [8]. The *bla*_EBC_ (5.8%; 3/52) identified from ESCR *E. coli* has been reported in the United Kingdom [39]. Plasmid-mediated AmpC-KP has been isolated from dogs and/or cats in South Korea [10], China [40,41], Japan [7], Switzerland [42], and Italy [3], and most of these belong to the CMY and DHA groups. In this work, no AmpC-KP isolates were also confirmed as ESBL producers, and vice versa. This might be related to the antimicrobials used in the treatment of animals [43]. In a previous study, CTX-M-2- and CMY-2-producing *E. coli* were reported in broiler chickens in Japan [44]. In addition, the susceptibility to carbapenems could be decreased by combination of AmpC production and porin deficiency [18]. Nevertheless, no ESBL/AmpC-KP showed activity as carbapenemase producers in this study.

ESBL and pAmpC-producing bacteria mostly have co-resistance with other antimicrobials [3,38]. The ESBL/AmpC genes are frequently located on an MDR plasmid, which plays a key role in their dissemination [45]. Our results showed the occurrence of MDR ESBL/AmpC-KP isolated from horses (3.3%; 7/212) was lower than from dogs and cats (30.1%; 31/103) in Japan [7]. Most MDR ESBL/AmpC-KP isolates showed co-resistance with aminoglycoside (*strA*- and *strB*-positive), tetracycline (*tetA*- and/or *tetB*-positive), and FOM. Only ESBL-KP isolates showed resistance to CP, but the *floR* gene, which is responsible for CP resistance, was detected in most CP non-susceptible AmpC-KP isolates. Similar to our results, MDR ESBL/AmpC-KP against aminoglycosides, tetracyclines, and amphenicol-mediated *strA/B*, *tet*, and *cat* genes have also been confirmed from dogs and cats in Italy [3]. Co-selection, when using antimicrobials other than ESCs for therapy, may maintain the existence of MDR ESBL/AmpC-producing bacteria in animals [38]. Treatment options for MDR ESBL/AmpC-KP infection might be limited when considering that several clinical cases have been reported from this species in horses. 

MLST analysis showed that *K. pneumoniae* ST-133 was identified as an ESBL producer in this study. Previously, ESBL-KP ST-133 has been reported in humans in Japan [46]. Four different STs of AmpC-KP (ST-145, ST-4830, ST-4831, and ST-4832) in this study have not been reported between humans and animals in Japan. AmpC-KP ST-145 and three new STs, which are SLV of ST-145, were only distributed at the JRA Ritto Training Center. Further investigation is needed to confirm whether the dissemination of ESBL/AmpC-KP occurred inside or outside the training center.

## 5. Conclusions

In conclusion, this is the first study that has shown *K. pneumoniae* carrying MDR pAmpC isolated from racehorses. Interestingly, our results showed that the percentage of pAmpC-KP is higher than ESBL-KP, as compared with other previous reports. Dissemination of MDR ESBL/AmpC-KP through fecal material in the training centers requires special attention among the racehorse community, as indirect transmission may occur in the environment. Risk of infection by MDR ESBL/AmpC-KP may occur in people who work in close contact with racehorses (e.g., veterinarians, caretakers, and owners).
